# Avocado Fruit on Postprandial Markers of Cardio-Metabolic Risk: A Randomized Controlled Dose Response Trial in Overweight and Obese Men and Women

**DOI:** 10.3390/nu10091287

**Published:** 2018-09-12

**Authors:** Eunyoung Park, Indika Edirisinghe, Britt Burton-Freeman

**Affiliations:** Center for Nutrition Research, Institute for Food Safety and Health, Illinois Institute of Technology, Chicago, IL 60616, USA; epark4@iit.edu (E.P.); iedirisi@iit.edu (I.E.)

**Keywords:** hass avocado, glycemic response, endothelial function, flow mediated vasodilation, lipoprotein particles, monounsaturated fat

## Abstract

Avocados are distinctive fruits having both fats and fibers along with various micronutrients and bioactive phytochemicals. This study aimed to assess the effects of replacing carbohydrate energy in meals with half or whole avocado on postprandial indices of metabolic and vascular health. A single-center, randomized, controlled, 3-arm, 6 h, crossover study was conducted in overweight/obese middle-aged adults (*n* = 31). Participants consumed energy-matched breakfast meals containing 0 g (Control), 68 g (Half-A) or 136 g (Whole-A) fresh Hass avocado on 3 separate occasions. Post-meal glycemic (*p* < 0.0001), insulinemic (*p* < 0.0001) and flow mediated vasodilation (FMD) responses were reduced compared to Control meal (*p* < 0.01), independent of dose. Nuclear magnetic resonance analyses indicated lower concentrations of triglyceride-rich lipoproteins and higher concentrations of larger high-density lipoprotein (HDL) particles after the Whole-A vs. the Control meal (*p* = 0.02, *p* < 0.05, respectively). Race/ethnicity influenced sub-class lipoprotein concentrations (*p* < 0.05). Oxidized low-density-lipoproteins, monocyte chemoattractant protein-1, and interleukin-6 were not different among meals. Tumor necrosis factor-α tended to be lower after Whole-A vs. Control meal (*p* = 0.07). Replacing carbohydrate components with avocados in a meal improved FMD, a measure of endothelial function, and improved glycemic and lipoprotein profiles in overweight/obese adults. The study provides insight on the acute cardio-metabolic benefits of incorporating avocados into a meal.

## 1. Introduction

It is well appreciated that diet has a significant role in disease development and protection. More recently, the quality of carbohydrates and fats has been under scrutiny for their impact on health. Epidemiologic studies have revealed that consumption of refined carbohydrate-containing diets and/or diets high in saturated fat (SFA) is associated with increased risk for coronary heart disease [1] and increased prevalence of type 2 diabetes [2]. Interventional trials have shown that consumption of relatively high amounts (52–62% carbohydrate energy) of refined carbohydrates induces adverse effects on glycemic [3] and blood lipid regulation [4,5] and regular intake of SFA and trans fats have been shown to increase total and low-density lipoprotein (LDL) cholesterol [6,7]. Accordingly, diets containing unwarranted amounts of refined carbohydrates and SFA are considered risk factors in developing metabolic and glycemic disturbances making them targets for control.

Saturated fats have been of cardio-metabolic risk concern for years, while diets containing higher amounts of monounsaturated fatty acid (MUFA) are suggested to be protective and have beneficial effects, such as enhancing insulin sensitivity [8] and improving plasma total and LDL-cholesterol levels [6,7]. Likewise, dietary patterns, including the Dietary Approaches to Stop Hypertension (DASH) diet, Mediterranean diet, and the Seventh-day Adventists diet, which emphasize eating more fruits and vegetables, nuts, whole grains, MUFA oils and fish, and have consistently been associated with health benefits [9,10,11]. In the last update of the Dietary Guidelines for Americans (2015–2020), recommendations highlighted eating nutrient dense foods and suggested that replacing SFA with complex carbohydrates, polyunsaturated (PUFA) and or MUFA could benefit total cholesterol and LDL-cholesterol concentrations [12]. Hence, dietary patterns emphasizing certain dietary components, such as MUFA, while decreasing other components, such as SFA or refined, readily digestible carbohydrates, may have favorable effects on cardio-metabolic health. From the consumer perspective, ease of exchange is critical when making dietary modifications; and single foods that inherently contain these dietary components are ideal for helping consumers meet heart and metabolically healthy goals.

Avocados are a distinctive fruit characterized by their nutrient profile containing MUFA, PUFA, dietary fiber, folate, potassium and several essential micronutrients and bioactive phytochemicals [13]. A recent clinical trial reported that consuming a diet containing one avocado a day for 5 weeks significantly reduced plasma LDL-cholesterol and small dense LDL-particle concentrations [14]. Furthermore, it has been demonstrated that consuming avocados within the context of a typical Western meal had favorable effects on glucose homeostasis [15] and insulin responses [16].

The typical Western meal is high in available carbohydrate and/or fat and has consistently been shown to disturb cardio-metabolic sequences including changes in lipid/lipoprotein metabolism, glucose homeostasis and insulin responsiveness [17]. Furthermore, exaggerated metabolic responses are detrimental to endothelial function via increased reactive oxygen species (ROS), vascular and intracellular adhesion molecules, pro-inflammatory mediators and vascular permeability [18,19]. These effects are apparent acutely (i.e., in the postprandial state) and chronically [20,21]. Managing day to day acute oxidative-immuno-metabolic imbalances that can result in cellular damage and dysfunction of tissues, such as the endothelium is an important strategy in reducing risk for the development and progression of cardio-metabolic diseases [22,23,24,25].

Therefore, the aim of the present study was to investigate the strategy of incorporating avocados into a breakfast meal replacing mainly carbohydrates with fat by adding MUFA and PUFA fats from avocados on postprandial indices of metabolic and vascular health. Endpoints of interest included changes in glycemic indices along with changes in endothelial function as measured by flow-mediated dilation (FMD) and biomarkers of inflammation and oxidative stress/damage.

## 2. Materials and Methods

### 2.1. Subjects and Consort Flow Diagram

**Figure 1:**
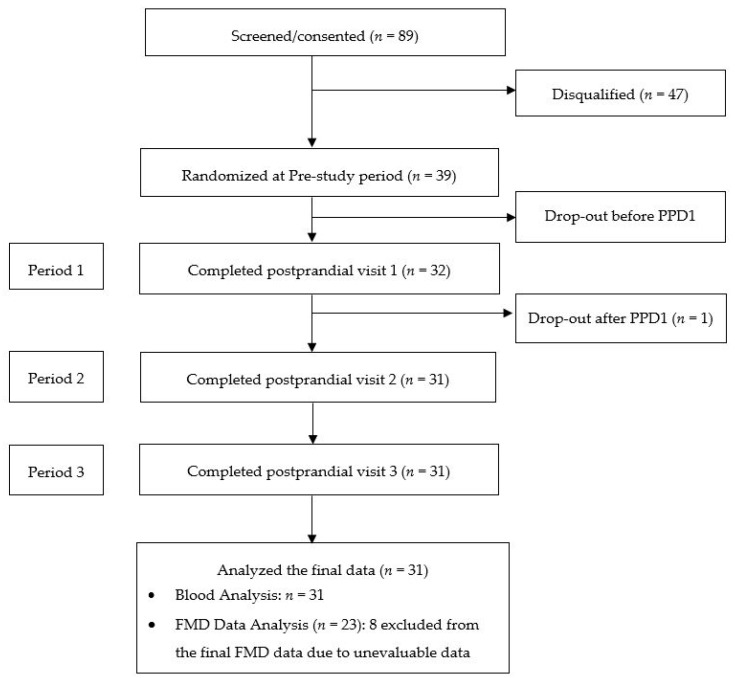
Consolidated Standards of Reporting Trials (CONSORT) flow diagram of the study. PPD, Postprandial study day; FMD, Flow mediated vasodilation.

Thirty-nine subjects (21 men and 18 women) were enrolled and randomized in the study; 31 subjects (15 men and 16 women) completed all protocol specified procedures (Figure 1).

### 2.2. Inclusion Criteria

Eligible subjects were non-smoking men and women with body mass index (BMI) between 25 and 35 kg/m^2^, with elevated fasting glucose (5.0–6.4 mmol/L) and insulin (no greater than 90.3 pmol/L) concentrations, and between 25 and 60 years of age. Individuals also had to have no clinical evidence or documented history of cardiovascular, respiratory, renal, gastrointestinal, or hepatic disease to be eligible.

### 2.3. Exclusion Criteria

Subjects who reported following a vegan diet, had unusual dietary habits (e.g., pica), were actively losing weight or trying to lose weight, addicted to drugs or alcohol or smoked or who were taking over the counter supplements (i.e., anti-oxidant, anti-inflammatory) or prescription medications (i.e., lipid-lowering or blood pressure lowering medications) or presented with significant psychiatric or neurological disturbances—all of which may interfere with study procedures or endpoint evaluation—were not eligible for participation. Subjects who consumed three or more servings of nuts per week were also excluded. Past smokers were allowed in the study if cessation > 2 years. Each subject was studied once.

### 2.4. Study Design

The trial is a single-center, randomized, single-blinded, diet-controlled, 3-arm, 6 h postprandial, crossover study performed at the Clinical Nutrition Research Center (CNRC) at the Illinois Institute of Technology (Illinois Tech, Chicago, IL, USA). The study was conducted according to the guidelines laid down in the Declaration of Helsinki and the International Conference on Harmonization-Good Clinical Practice (ICH-GCP) and all procedures involving human subjects were approved by the Institutional Review Board at the Illinois Institute of Technology (IRB2015-001). Written informed consent was obtained from all subjects before the initiation of the study. The study was conducted from 2015 to 2016 in Chicago, IL, USA. This trial was registered at clinicaltrials.gov as NCT02479048.

### 2.5. Study Timeline and Procedures

**Figure 2:**
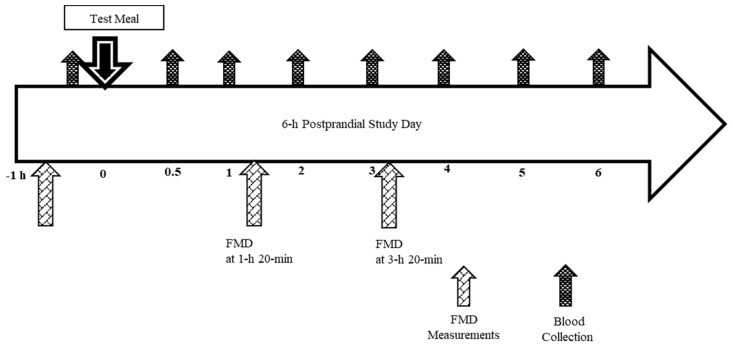
6-h postprandial study day schema. FMD indicates flow mediated vasodilation.

All qualified subjects participated in a pre-study visit that included instruction on completing study questionnaires, 24 h dietary recalls (the National Cancer Institute ASA24^®^ Recall online, the National Cancer Institute, Bethesda, MD, USA), and strategies for restricting avocado and olive oil intake and colored plant foods rich in phytonutrients 3 days prior to each postprandial test day visit (PPD). In addition, subjects were asked to restrict intake of alcohol, coffee, tea, and other caffeinated beverages and limit their physical activity 24 h prior to a testing day. Main dish (one frozen Stuffer’s Entrée, ~270 Kcals) and soft drinks (Lemonade or Ginger ale ~270 kcals) the night before each testing day were provided and subjects chose side dishes at home, which were monitored to maintain consistency among visits. Additionally, subjects were asked to maintain usual dietary intake and sleep patterns and not consume anything except for water after 10 pm the night before a testing day. Each testing day started with confirming adherence to the study-specific protocol (diet, exercise, sleep, and fasting status). Thereafter, body weight and blood pressure were measured and baseline FMD assessment performed. After the FMD, an intravenous catheter was placed and baseline blood sample collected. Next, subjects received one of the three test breakfast meals according to the randomization schedule and were asked to finish eating within 20 min (Figure 2). Blood samples were collected at 0 h (fasting, baseline) and 0.5 h, 1 h, 2 h, 3 h, 4 h, 5 h, and 6 h starting timers at the first bite of breakfast (Figure 2). FMD was measured at 1 h 20 min and 3 h 20 min. After the final 6 h blood collection, catheters were removed and subjects were prepared for their next visit, repeating instructions from the pre-study visit and confirming the date and time of the next scheduled PPD. To minimize variability due to female hormone fluctuations, women were studied in the follicular phase of their menstrual cycle. For this purpose, time between study visits could be up to 4 weeks.

### 2.6. Test Meals

Three meals were prepared: two meals contained either 68 g fresh Hass avocado (1/2 avocado; Half-A) or 136 g fresh Hass Avocado (1 avocado; Whole-A) and one meal (the Control meal) did not contain avocado (Table 1 and Table 2). Avocado meals aimed to replace some of the carbohydrate components in the Control meal while maintaining similar energy content. All meals were prepared in the metabolic kitchen at the CNRC under the supervision of the registered dietitian following strict food safety standards. Subjects came to the laboratory on three separate occasions. Subjects consumed each meal once based on a randomly assigned sequence by computer-generated randomization allocation list.

### 2.7. Height and Weight

Height was measured to the nearest 0.1 cm with the use of a wall-mounted stadiometer. Weight (light clothing only) was measured to the nearest 0.1 kg with the same digital scale each time. The scale was calibrated biweekly. These data were used to derive body mass index (BMI, kg/m^2^).

### 2.8. Metabolic Responses

Blood was collected from indwelling catheters at protocol-specified time points into Ethylenediaminetetraacetic acid (EDTA) tubes and centrifuged at 12,857× *g* at 4 °C for 15 min to obtain plasma. Aliquots of plasma were stored immediately at −80 °C for subsequent analysis. Plasma glucose concentrations were measured using standardized enzyme-based assay kits (Cat # TR3823 and GL3815, respectively, Randox, Antrim, UK) on the Randox Daytona Auto Clinical Analyzer (Randox, Antrim, UK). Plasma insulin was assessed using an immunoturbidimetry assay (Cat# KAI071, Kamiya Biomedicals, Tukwila, WA, USA). Plasma oxidized low density lipoprotein (Ox-LDL) was measured using ELISA assay kits (Cat# 10-1143-01, Mercodia Inc., Winston Salem, NC, USA). Interleukin (IL)-6 and Monocyte chemoattractant protein (MCP)-1 in plasma samples were measured using high sensitive ELISA assay methods (Cat# HS 600B and DCP00 respectively. R&D Systems, Minneapolis, MN, USA). All assay protocols were performed according to the manufacturers’ instructions and appropriate quality controls were used as applicable. Intra and inter assay % coefficient of variation (CV) was below 10% in all the assays tested. Lipoprotein particles were analyzed for two meals (Control and Whole avocado meals) using Nuclear magnetic resonance (NMR) spectra of frozen plasma specimens by LipoScience (Raleigh, NC, USA).

### 2.9. Flow Mediated Dilation (FMD)

Flow mediated vascular reactivity was studied in the brachial artery using methods recommended by the American College of Cardiology [26]. Briefly, the brachial artery was imaged by ultrasound (GE LOGIQ e 2008, General Electric Healthcare, Wauwatosa, WI, USA) and resting measurements of vessel diameter (mm) collected. Thereafter, a blood pressure cuff was applied below the elbow and inflated to a pressure of 220 mmHg systolic pressure for 5 min. Immediately after cuff deflation, brachial artery vessel diameter was monitored and measured to obtain peak vessel relaxation. Ultrasound measurements were recorded as a series of moving video images for 2 min. Brachial artery diameters were measured at end-diastole determined by ECG-triggered gating procedures using Brachial Analyzer for Research (Medical Imaging Applications-LLC, Coralville, IA, USA).

### 2.10. Sample Size

Based upon previous studies [27,28], a sample size of 24 completers was calculated for detecting a 12% difference in 2 h incremental area under the curve (iAUC) for glucose with a power of >80%. 25% attrition rate was included in enrollment plan to meet sample size requirements.

### 2.11. Statistical Methods

Subject characteristics were analyzed and tabulated using descriptive statistics from data collected at the screening visit. Results were presented as numbers and percentages, as appropriate. Shapiro-Wilk tests, skewness, and kurtosis were used to assess normality for continuous variables. Data not conforming to normal distribution patterns were log transformed prior to analysis and noted accordingly. Outlier removal or equipment malfunction (ultrasound) may have resulted in fewer evaluable subjects for secondary endpoints and were indicated accordingly. Mixed-model analysis of repeated measures was performed on each quantitative outcome variable to test main effects of 3 meals (Control meal, Half-A, and Whole-A) and time (h) using PROC MIXED via Window PC-SAS (version 9.4; SAS Institute Inc., Cary, NC, USA). In the final analyses, meal and time were included and the corresponding baseline value was included as a covariate (*p* < 0.0001) and the Kenward-Roger correction and the method of restricted maximum likelihood were used in all Mixed Models [29,30,31]. Multiple comparisons within and among meals over 6 h postprandial time were performed by mixed model statistical significance (*p* < 0.05). Postprandial peak concentrations of metabolic markers were analyzed. The results of the statistical analysis were presented as least square means (LSM) ± SEMs unless indicated otherwise. Statistical significance was based on 2-sided meal comparison at the 5% significance level under a null hypothesis of no difference between meals.

## 3. Results

### 3.1. Subjects

Thirty-nine subjects were enrolled into the study. Thirty-one subjects completed all three study visits (Figure 1). Baseline characteristics of subjects completing all 3 postprandial study visits are presented in Table 3. All study foods were well liked and tolerated and no adverse events related to study foods were reported during the study. 

### 3.2. Glucose and Insulin

**Figure 3:**
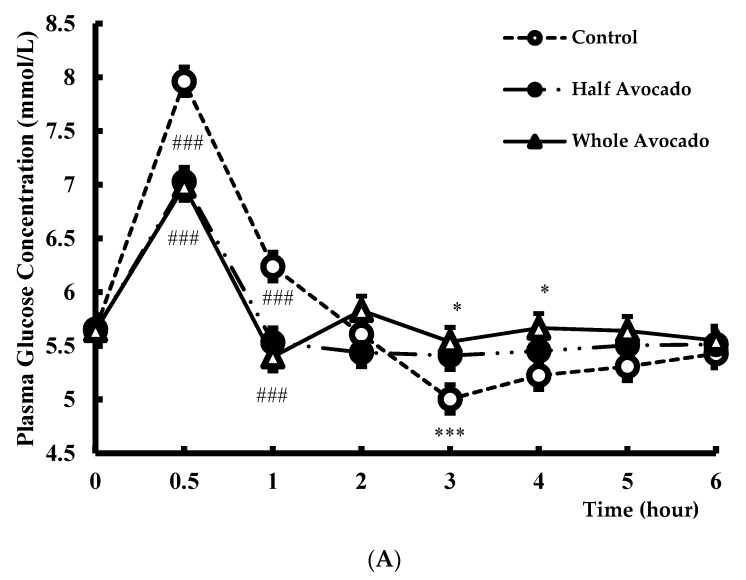

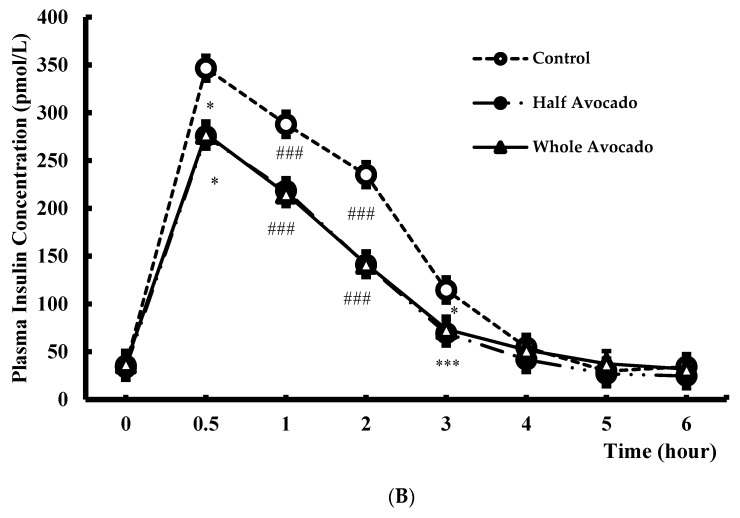
(**A**) Plasma glucose response over 6 hours after consuming test meal. The figure legend indicates test meal groups (*n* = 31). Data were analyzed by PROC MIXED using SAS 9.4. Main effects of meal *p* = 0.07, time *p* < 0.0001, and meal by time *p* < 0.0001. Data are means ± SE, *n* = 31. * *p* < 0.05, *** *p* < 0.005, ### *p* < 0.0001 for the difference compared to Control; (**B**) Plasma Insulin response over 6 h after consuming test meal. The figure legend indicates test meal groups (*n* = 31). Data were analyzed by PROC MIXED using SAS 9.4. Main effects of meal *p* < 0.0001, time *p* < 0.0001, and meal by time *p* < 0.0001. Data are means ± SE, *n* = 31. * *p* < 0.05, *** *p* < 0.005, ### *p* < 0.0001 for the difference compared to Control.

Consuming test meals containing either half or full avocado (Half-A and Whole-A) significantly decreased the postprandial glycemic and insulinemic responses to meals over 6 h compared to the Control meal (both *p* < 0.0001), including 2 h iAUC (*p* < 0.0001). Postprandial glucose peak concentrations were also significantly lower after consuming both avocado-containing meals (7 mmol/L ± 0.2) compared to the control meal (8.1 mmol/L ± 0.2, *p* < 0.0001). Similar effects were observed in postprandial insulin peak concentrations after both avocado-containing meals compared to the Control meal (*p* < 0.005). No differences between the Half-A and Whole-A meals on glucose and insulin were observed (Figure 3A,B).

### 3.3. Flow Mediated Vasodilation (FMD)

**Figure 4:**
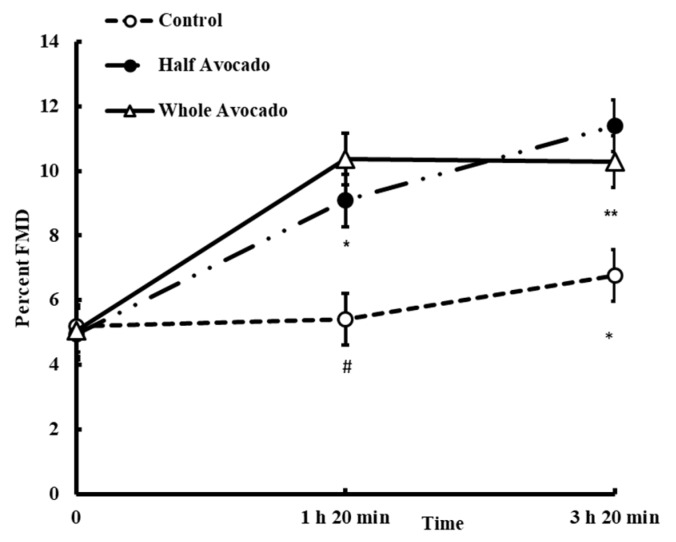
Flow-mediated vasodilation responses over 6 hours after consuming study meal. The figure legend indicates test meal groups (*n* = 23). Data were analyzed by PROC MIXED using SAS 9.4. Main effects of meal *p* =0.0002, time *p* < 0.0001, and meal by time *p* = 0.0018. Data are means ± SE, *n* = 23. * *p* < 0.05, ** *p* < 0.01, # *p* < 0.001 for the difference compared to Control.

Changes in FMD were not different between meals at baseline (*p* > 0.05); however significant effects of meal (*p* = 0.0002), time (*p* < 0.0001) and meal by time interaction were evident (*p* = 0.002, Figure 4). In general, after eating the Control meal, FMD increased ~0.2% and ~1.6% at 1 h 20 min and 3 h 20 min in later, respectively, whereas when avocado was incorporated in the meals, percent FMD doubled from ~5% (at time 0 min) to ~10% at 20 min past 1 and 3 h. Both the Half-A and Whole-A meals significantly increased FMD compared to the Control meal at 1 h 20 min (*p* = 0.0346, *p* = 0.0007, respectively) and at 3 h 20 min (*p* = 0.0013, *p* = 0.045, respectively).

### 3.4. Plasma Lipoprotein Particle Number and Size Using Nuclear Magnetic Resonance (NMR)

NMR analyses of lipids/lipoprotein particles were measured at 0, 2, 4, 6 h. Mean differences between meals over the 6 h period are shown in Table 4 and changes over time shown in Supplemental Figures S1–S8. Total plasma TG did not differ significantly between meals (*p* = 0.09, Control meal 1.18 mmol/L ± 0.04 and Whole-A meal 1.15 mmol/L ± 0.04), NMR analyses revealed significantly lower TG-rich lipoproteins (total chylomicron and very-low-density lipoprotein (VLDL) concentrations) after the Whole-A meal compared to the Control meal (Table 4, *p* = 0.02), with the greatest difference observed at 6 h (Supplement Figure S1). Over the course of the 6 h postprandial period, the mean concentration of total LDL particles did not change, but overall tended to be higher after the Whole-A meal compared to the Control meal (*p* = 0.07), due to a significant decrease at 2 h after the Control meal (Supplement Figure S2). The concentration of different sizes of circulating particles showed relatively higher concentrations of small LDL particles (nmol/L) after the Whole-A meal compared to the Control meal (*p* = 0.009, Table 4 and Supplemental Figure S3) evident at 2 and 4 h post-meal, with intermediate density lipoprotein (IDL) lower after the Whole-A meal compared to Control meal (*p* = 0.004) and no difference between meals for large LDL particles (Table 4 and Supplement Figures S4 and S5). Alternatively, the concentration of large and medium HDL particles was higher after the Whole-A compared to Control meal (*p* = 0.06 and *p* = 0.004, respectively, Table 4 and Supplement Figures S6 and S7). Lower concentrations of the small HDL particles were also observed after the Whole-A vs. control meal (*p* = 0.009, Table 4 and Supplement Figure S8). Mean particle sizes (nm) were not different between meals.

Testing for effects of BMI, age, sex, race/ethnicity on meal related lipoprotein outcomes indicated a significant effect of race/ethnicity on both LDL and HDL particle size concentrations (*p* = 0.004 and *p* = 0.0003, respectively). Albeit small sample size the data are worth noting for future investigation: Lower concentrations of small LDL particles were observed after the Whole-A meal compared to the Control meal in all groups except those individuals who qualified themselves as Asian (*p* < 0.05, Supplemental Table S1). Likewise, the Asian group had lower concentrations of the large HDL particles after the Whole-A meal, whereas the Caucasian group had higher concentrations of the larger HDL particles after the Whole-A meal compared to the Control meal (*p* < 0.05). Correspondingly, lower concentrations of the small HDL particles after the Whole-A meal compared to the Control meal were apparent in the Caucasian and Hispanic groups. Mean particle sizes (mm) were consistent with these findings.

### 3.5. Inflammatory and Oxidative Stress Markers

Increases in IL-6 were observed post-meal, but not different between meals. Plasma concentrations of MCP-1 were also not different between meals. Alternatively, tumor necrosis factor alpha (TNF-α) tended to be lower after the Whole-A meal compared to the Control meal (*p* = 0.07). No differences in the concentration of Ox-LDL were observed over time or between meals.

## 4. Discussion

The aim of the present study was to investigate the strategy of incorporating avocados into a breakfast meal by replacing mainly carbohydrate components with avocado fats (mainly MUFA and PUFA) on postprandial indices of metabolic and vascular health. Endpoints of interest included changes in glycemic indices, changes in endothelial function as measured by FMD, and biomarkers of inflammation and oxidative stress/damage. The results demonstrated reduced postprandial glycemia, including reduced peak glucose and insulin concentrations, along with increased vaso-relaxation when either a half or whole avocado was included in the meal compared to the Control meal with no avocado.

Since the late 1970s, beginning with observations by Zilversmit [32], evidence has accumulated indicating a relationship between postprandial (dys)metabolism, specifically hyperglycemia and cardiovascular disease risk, and this relationship extends to people without diabetes [33]. The American Diabetes Association stated that postprandial plasma glucose concentrations peak ~1 h, rarely exceed 7.8 mmol/L and return to baseline level within 2–3 h for people without diabetes [34]. Additionally, the European Diabetes Policy Group (EDPG) has set targets for postprandial peak glucose concentrations less than 7.5 mmol/L to reduce arterial risk and 8.9 mmol/L to lower microvascular risk [35]. Among the consequences of acute glycemia are impaired endothelial function [36], promotion of an acute inflammatory response [37] and induction of a hyper-coagulable state [38]. In vitro studies examining effects of hyperglycemia suggest many of the negative consequences are from overproduction of superoxide radicals from the electron transport chain in the mitochondria [39]. Therefore, dietary strategies to reduce postprandial hyperglycemia are prudent in cardiovascular health, even in the non-diabetic population. Our results demonstrated replacing carbohydrates in a meal with half or a whole avocado dropped peak glucose to levels within a relatively normal range [34] and below the threshold of 7.5 mmol/L suggested by EDPG [35]. Peak insulin concentrations were also lower after consuming meals with avocado suggesting reduced burden on the pancreas while managing glucose concentrations. Moreover, replacing carbohydrates with avocados guarded against exaggerated drops in glucose concentrations below baseline: drops which have been observed with glucose tolerance tests and standard high carbohydrate mixed meals [40], and was observed in the present study after the Control meal. Overall, managing glycemic responses to meals in a favorable range has important health implications for lowering diabetes and cardiovascular disease (CVD) risk [41]. From a practical standpoint, avocados are a relatively easy nutrient-rich dietary option to help manage post-meal glycemia.

Meals characterized as high in energy containing readily available carbohydrates and fat, typical of Western eating patterns, promote hyperglycemia and hypertriglyceridemia, elevate ROS [20,42] and promote endothelial dysfunction [43,44]. Taking these meal types as done in the present study and isocalorically exchanging mostly carbohydrate rich components to include avocadoes resulted in significantly improved endothelial function as measured by FMD. This favorable response was observed over time postprandially, and compared to the Control meal. The mechanism underlying the effect is not clear; however, others have shown vascular function effects with Mediterranean diets or MUFA-rich diets. Buscemi et al., (2009) described a significant improvement in FMD measured after fasting within the first week of following a Mediterranean diet (40% increase from baseline) in 10 overweight/obese participants, which was significantly different than FMD responses observed within a week of following the Atkins diet (FMD decreased by over 50%) [24]. FMD was also increased in abdominally obese adults following a Mediterranean diet for 2 months [25]. In another study, Choudhury et al. reported a significant increase in FMD after 4 weeks of snack replacement with 50 g almonds (a high MUFA snack) in 20 healthy young men, compared to subjects’ habitual diet [45]. Alternatively, some studies have demonstrated no effect of the Mediterranean diet [46] or MUFA (from olive oil)—rich diets on FMD [6]. In at least one study, olive oil intake has shown to reduce postprandial FMD by 31% in 10 healthy adults [47]. The inconsistent findings may be due to study design differences, such as acute vs. chronic interventions, fasting vs. postprandial evaluations, co-existing nutrients or bioactives, dosages, and study populations. Although the mechanistic underpinnings of the observed FMD effects in the current study cannot be explained specifically, the marked reductions in glycemia could have been a factor, possibly influencing local ROS production, nitric oxide (NO) bioavailability and/or other activity in the endothelium. The bioactive components in avocado may have also played a role. Our findings support a favorable effect of avocado on vascular endothelial function and support further research to identify the mechanisms of avocado-associated vascular actions.

Fasting and non-fasting lipid and lipoprotein metabolism was accessed by NMR. NMR lipoprotein results revealed that total plasma triglyceride concentrations were not statistically different between meals, the triglyceride-rich lipoprotein fraction (chylomicrons and VLDL) was lower after the Whole-A meal compared to the Control meal while the concentration of larger HDL particles were higher after Whole-A vs. Control meals. One interpretation is that triglycerides were being transferred from chylomicron and VLDL particles to HDL particles before clearance [48]. This lipoprotein pattern may be considered athero-protective [49]. Studies of HDL metabolism relative to cardiovascular risk suggest that greater number of larger HDL particles are inversely associated with decreased cardiovascular risk [49], particularly in light of the corresponding favorable FMD responses.

The Control meal lipoprotein results may be due to its low-fat, high carbohydrate composition that can promote increased plasma levels of liver-derived triglyceride-rich lipoproteins (VLDL). Elevated VLDL can give rise to remnant particles and small, dense LDL particles which are considered atherogenic. While there was a statistical trend (*p* < 0.05–0.1) toward higher concentrations of medium size chylomicron/VLDL and LDL/IDL particles and higher concentrations of small HDL particles after the Control meal (*p* = 0.009), the non-fasting small LDL particle concentrations were lower in the Control meal compared to the Avocado meal. These profiles could be due to the carbohydrate differences in meals and the marked increases in insulin concentrations after the Control meal corresponding with reduced small LDL concentrations at 2 h and 4 h, or an effect of higher total fat content in the avocado containing meal, specifically the higher SFA content, mostly from added butter to balance fat between Half and Whole avocado meals. SFA intake is associated with increasing concentrations of small LDL particles and MUFA have the opposite effect [50]. Wang et al., (2015) replaced SFA with MUFA from avocado and reported reduced fasting small, dense LDL cholesterol and increased average LDL particle size in a 5-week crossover intervention [14]. Another study showed lowered VLDL-C and increased apolipoprotein A–I, the major protein component of HDL particles, after replacement of high-carbohydrate diet with MUFA diet for 28 days [51]. There is also evidence that heritable genotypic effects influence diet-induced lipoprotein subclass changes [52]. Interestingly, we found that race/ethnicity significantly influenced meal-related lipoprotein particle size and concentration responses, particularly as it related to the Asian group responses. The Asian group had both lower concentrations of the large HDL particles and higher concentrations of the small LDL after the avocado meal whereas the opposite patterns were observed with other race/ethnic groups. Few studies have investigated the relationship between lipid/lipoprotein profiles and racial/ethnic differences [53,54]. Vega et al., showed that African-American had higher HDL-C concentrations compared to Caucasian, suggesting genetic differences in hepatic lipase activity [55]. The INTERHEART study found lower concentrations of LDL-C, HDL-C and triglyceride concentrations in East Asian compared to non-Asian individuals [56]. The data at present are mostly cross-sectional and do not examine sub-class profiles. Although the sample set for our race/ethnicity analysis was unmatched and not powered to make conclusions, our data combined with the available epidemiology suggest a need for further research to understand the race/ethnic disparity in diet therapy and how results might influence dietary recommendations for cardio-metabolic health.

Markers of oxidative damage and inflammation are known to respond acutely to meals, especially high fat and/or high-carbohydrate diet [20,42]. One randomized controlled trial has shown that MUFA diets from oleic acid can reduce IL-6, a marker of inflammation [57], while others have demonstrated no effect [58]. Intake of a high MUFA (~20% of energy) diet for 12 weeks reduced postprandial MCP-1 and TNF-mRNAs in peripheral blood mononuclear cell (PBMC) compared to a high SFA diet [59]. However, the current study did not find meal associated differences in biomarkers of inflammation or oxidative stress/damage. One reason may be because our study was designed as an acute evaluation of postprandial changes after a single meal. Longer-term feeding studies may provide alternative results.

The study had strengths and limitations. This was an acute postprandial study that allows for understanding effects of nutritional factors during the dynamics of metabolic processing. The study design does not allow for making conclusions about long term effects which will require follow up studies. The study was performed in an at risk population providing insight on the cardio-metabolic effects of dietary manipulations with avocados as a source of health-promoting nutrients. The study is relatively unique in that dietary substitutions with fat-rich foods typically focus on replacing SFA with MUFA whereas our study replaced carbohydrate with mostly MUFA. Additionally, the study replaced carbohydrates in a standard breakfast meal with two intake levels of avocados, while maintaining the energy content of the meals to minimize energy-associated variability in responses. Data comparisons with previous published work are difficult because of the lack of research in this area, particularly as it relates to lipoprotein remodeling postprandially and the influence of ethnic/racial diversity on clinical endpoints.

## 5. Conclusions

In conclusion, replacement of energy from carbohydrate with energy from avocados rich in MUFA, PUFA, fiber and bioactive phytochemicals showed beneficial effects on glycemic and vascular markers during an acute postprandial challenge in middle-aged, overweight/obese adults. Incorporating fresh Hass avocados in meals can help people achieve dietary recommendations to eat more fruits and vegetables and simple substitution strategies with avocados for carbohydrates can add to the nutrient diversity of the diet and potentially have important cardio-metabolic benefits worthy of investigating further.

## Figures and Tables

**Table 1:** Nutrient composition of breakfast meals for postprandial study visits *.

	Control Meal	Half Avocado Meal	Whole Avocado Meal
Energy (kcal)	637.4	617.6	642.3
Carbohydrate (g)	120.6	78.37	80.7
Sugar (g)	59.4	26.5	25.5
Fiber (g)	4.9	8.6	13.1
Protein (g)	18.4	18.2	16.7
Fat (g)	9.9	27.3	30.7
Saturated fat (g)	4.9	11.3	8.6
Trans fat (g)	0.3	0.6	0.3
Monounsaturated fat (g)	2.1	10.8	15.8
Polyunsaturated fat (g)	1.2	2.6	3.6

* Nutrients of food ingredients analyzed by Food Processor Pro SQL Edition by ESHA (Version 10.15.41, ESHA Research, Salem, OR, USA).

**Table 2:** Breakfast meal foods (grams, g) for postprandial study visits *.

Breakfast Items	Control Meal	Half Avocado Meal	Whole Avocado Meal
Hass Avocado, fresh	0	68	136
Bagel, plain ^†^	99	68	65
Cream Cheese, fat free ^‡^	40	55	35
Cucumber without skin	15	15	15
Romaine lettuce	10	10	10
Butter, unsalted ^§^	8	18	10
Honeydew melon	90	60	60
Instant oatmeal (maple and brown sugar) ^||^	25	25	25
Brown sugar	15	0	0
Unsweetened lemonade mix ^¶^	1	1	1
White sugar	20	5	5
Water ^«^	310	304	310

* All ingredients were purchased at a local grocery store in Chicago, Illinois and are presented in gram (g) amounts. ^†^ Thomas^®^ Bagels Plain Pre-Sliced, Bimbo Bakeries USA, Inc., Horsham, PA 19044, USA; ^‡^ Philadelphia Fat Free Cream Cheese. Kraft Foods, Inc., Northfield, IL 60093-2753, USA; ^§^ Ahold^®^ Unsalted Sweet Cream Butter. Ahold USA, Inc., Landover, MD 20785, USA; ^||^ Quaker^®^ Instant Oatmeal Maple and Brown Sugar. The Quaker Oats Company, Chicago, IL 60604-9003, USA. ^¶^ Kool-Aid Unsweetened Drink Mix Lemonade. Kraft Heinz Foods Company, Northfield, IL 60093-2753, USA. ^«^ Water used for oatmeal (30 g) and drinking.

**Table 3:** Demographic information (Mean ± SD) *^,†^.

Variable		Total Subjects (*n* = 31)
Age (year)		37.9 ± 10.3
BMI (kg/m^2^)		29.0 ± 2.4
Mid-point waist circumference (cm)		92.9 ± 10.1
Systolic blood pressure (mmHg)		110.7 ± 8.0
Diastolic blood pressure (mmHg)		73.9 ± 7.5
Venous fasting glucose concentration (mmol/L)		5.6 ± 0.4
Venous fasting insulin concentration (pmol/L)		60.4 ± 20.8
Race/Ethnicity, *n* (%)	Caucasian	9 (29)
	African-American	13 (42)
	Asian	5 (16)
	Hispanic	4 (13)
Gender, *n* (%)	Male	15 (48)
	Female	16 (52)
BMI (kg/m^2^) Categories ^‡^, *n* (%)	Overweight	23 (74)
	Obese I	8 (26)

* Abbreviations: BMI, body mass index; kg, kilogram; m, meter; cm, centimeter; mmol/L, millimoles per liter; pmol/L, picomoles per liter. ^†^ Data was obtained from screening visit and presented as mean ± SD for *n* = 31 completers. ^‡^ Overweight was defined as 25 ≤ BMI (body mass index) < 30; Obese I, 30 ≤ BMI < 35.

**Table 4:** Effect of breakfast meals on lipoprotein variables by nuclear magnetic resonance, NMR *^,†^.

	NMR Analysis (Variable)	Control Meal	Whole Avocado Meal	*p* Value Con vs. Whole-A
Chylomicron/VLDL Particle concentration (nmol/L)	Total	49.2 ± 1.2	46.1 ± 1.3	0.02
	Large	3.5 ± 0.1	3.7 ± 0.1	0.36
	Medium	14.6 ± 0.8	13.3 ± 0.8	0.07
	Small	31.2 ± 1.3	29.0 ± 1.3	0.12
LDL Particle concentration (nmol/L)	Total	962.5 ± 15.3	986.7 ± 15.7	0.07
	LDL, Large	276.3 ± 9.7	277.8 ± 10.0	0.87
	LDL and IDL, Medium	232.2 ± 9.8	214.5 ± 10.2	0.11
	LDL, Small	456.0 ± 12.5	492.8 ± 13.0	0.009
HDL Particle concentration (nmol/L)	Total	35.0 ± 0.2	35.2 ± 0.2	0.54
	Large	8.2 ± 0.8	8.4 ± 0.8	0.06
	Medium	12.8 ± 0.3	13.9 ± 0.3	0.004
	Small	13.9 ± 0.3	12.9 ± 0.4	0.009
Average Particle size (mm)	VLDL	47.8 ± 0.5	48.5 ± 0.5	0.17
	LDL	20.7 ± 0.4	20.7 ± 0.4	0.94
	HDL	9.6 ± 0.01	9.6 ± 0.02	0.09

* All values for each variable represent mean ± SEM (*n* = 31). ^†^ Abbreviation: Con, Control meal; HDL, high-density lipoprotein; LDL, low-density lipoprotein; VLDL indicates very-low-density lipoprotein; Whole-A, Whole Avocado meal.

## Supplementary Materials

The following are available online at http://www.mdpi.com/2072-6643/10/9/1287/s1, Table S1: Lipoprotein metabolism using NMR analyses by racial/ethnic group, Figure S1: Plasma total chylomicron/VLDL particle concentrations over 6 h after consuming test breakfast meals, Figure S2: Plasma total LDL particle concentrations over 6 h after consuming test breakfast meals, Figure S3: Plasma small LDL particle concentrations over 6 h after consuming test breakfast meals, Figure S4: Plasma IDL particle concentrations over 6 h after consuming test breakfast meals, Figure S5: Plasma large LDL particle concentrations over 6 h after consuming test breakfast meals, Figure S6: Plasma large HDL particle concentrations over 6 h after consuming test breakfast meals, Figure S7: Plasma medium HDL particle concentrations over 6 h after consuming test breakfast meals, Figure S8: Plasma small HDL particle concentrations over 6 h after consuming test breakfast meals.
