# Supplementary material for: Avocado Fruit on Postprandial Markers of Cardio-Metabolic Risk: A Randomized Controlled Dose Response Trial in Overweight and Obese Men and Women
Source: Nutrients. 2018 Sep 12;10(9):1287. doi: 10.3390/nu10091287 (PMC6164649; doi:10.3390/nu10091287)
Supplement: Supplementary file 1 [file nutrients-10-01287-s001.pdf]

SUPPLEMENTAL MATERIALS

Intended for publication

Table S1. Lipoprotein metabolism using NMR analyses by racial/ethnic group*

NMR Analysis	Racial/Ethnic group	Control Meal	Whole-Avocado Meal	**P value Con vs Whole-A**
Large HDL Particle Concentration (nmol/L)	African-American	8.3 ± 0.1	8.5 ± 0.1	0.37
	Asian	8.6 ± 0.2	8.0 ± 0.2	0.005
	Hispanic	7.8 ± 0.2	8.2 ± 0.2	0.07
	Caucasian	8.1 ± 0.1	8.6 ± 0.1	0.001
Small HDL Particle Concentration (nmol/L)	African-American	14.1 ± 0.5	13.5 ± 0.6	0.27
	Asian	13.2 ± 0.9	14.4 ± 0.9	0.21
	Hispanic	13.7 ± 1.0	10.6 ± 0.9	0.003
	Caucasian	14.1 ± 0.6	12.5 ± 0.6	0.02
Small LDL Particle Concentration (nmol/L)	African-American	457.0 ± 20.0	502.9 ± 21.3	0.04
	Asian	409.6 ± 32.9	548.1 ± 33.2	<0.0001
	Hispanic	484.8 ± 36	477.3 ± 35.4	0.84
	Caucasian	468.1 ± 23.6	458.3 ± 23.9	0.69
Average HDL Particle Size (nm)	African-American	9.6 ± 0.02	9.1 ± 0.02	0.04
	Asian	9.7 ± 0.04	9.5 ± 0.04	<0.0001
	Hispanic	9.6 ± 0.04	9.6 ± 0.04	0.18
	Caucasian	9.5 ± 0.03	9.6 ± 0.03	0.001
Average LDL Particle Size (nm)	African-American	20.8 ± 0.1	20.7 ± 0.1	0.10
	Asian	20.8 ± 0.1	20.7 ± 0.1	0.31
	Hispanic	20.7 ± 0.1	20.8 ± 0.1	0.28
	Caucasian	20.6 ± 0.1	20.8 ± 0.1	0.04

*All values for each variable represent mean ± SEM (n=31): African-American, n=13; Asian, n=5; Hispanic, n=4; Caucasian, n=9).

Con, Control meal; HDL, high-density lipoprotein; LDL, low-density lipoprotein; VLDL, very-low-density lipoprotein; Whole-A, Whole Avocado meal

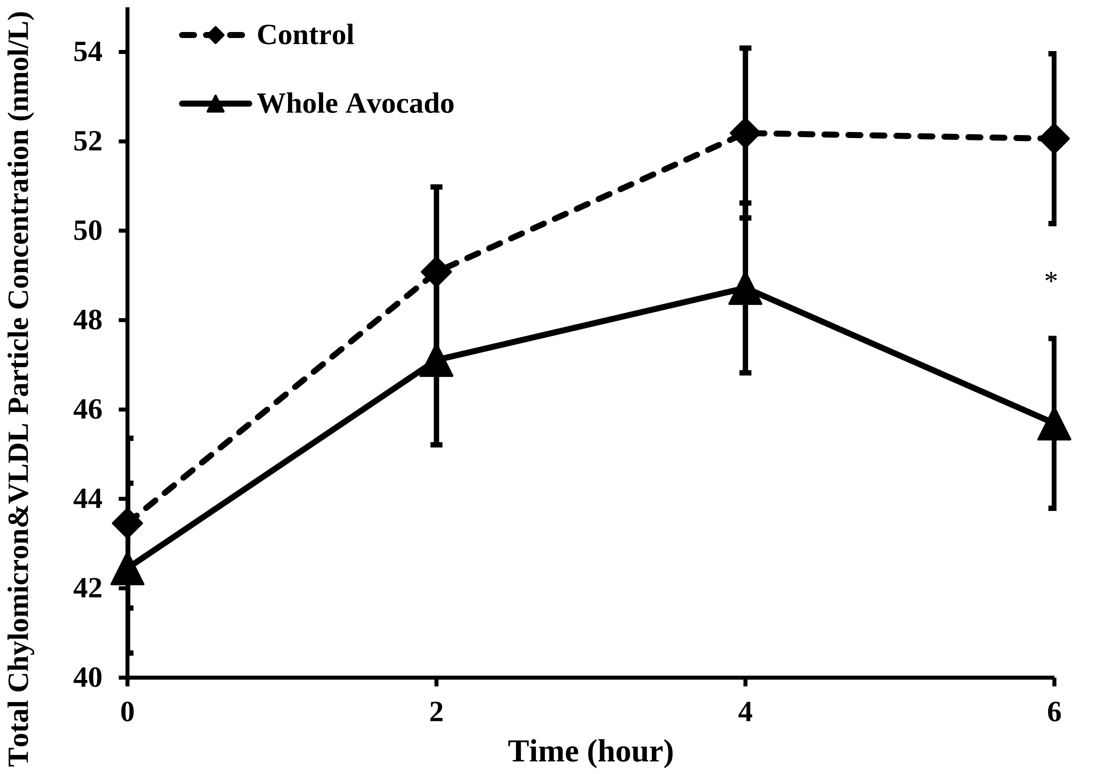

Figure S1. Plasma total chylomicron/VLDL particle concentrations over 6 h after consuming test breakfast meals (n=31). Data were analyzed by PROC MIXED using SAS 9.4. Main effects of meal *p* =0.009, time *p*=0.0001, and meal by time p=0.434.

Data are means ± SE, n=31. * p<0.05 for the difference compared to the Control meal.

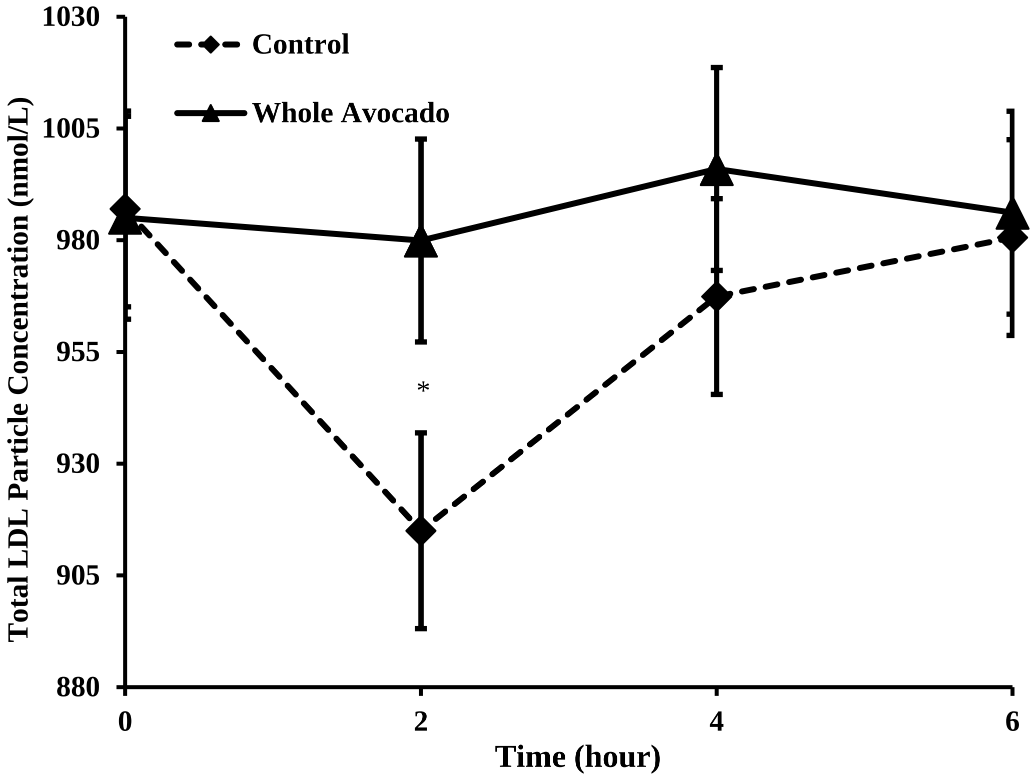

Figure S2. Plasma total LDL particle concentrations over 6 h after consuming test breakfast meals (n=31). Data were analyzed by PROC MIXED using SAS 9.4. Main effects of meal *p* =0.07, time *p*=0.13, and meal by time p=0.278.

Data are means ± SE, n=31. * p<0.05 for the difference compared to the Control meal.

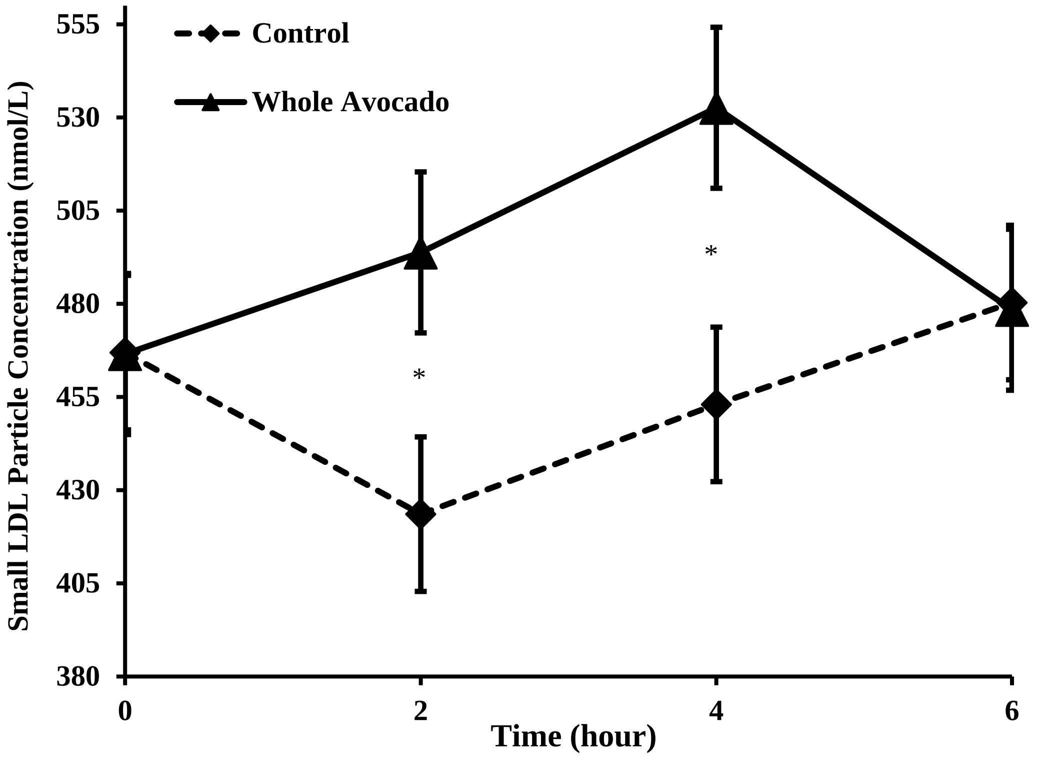

Figure S3. Plasma small LDL particle concentrations over 6 h after consuming test breakfast meals (n=31). Data were analyzed by PROC MIXED using SAS 9.4. Main effects of meal p =0.009, time p=0.32, and meal by time p=0.06.

Data are means ± SE, n=31. * $p<0.05$ for the difference compared to the Control meal.

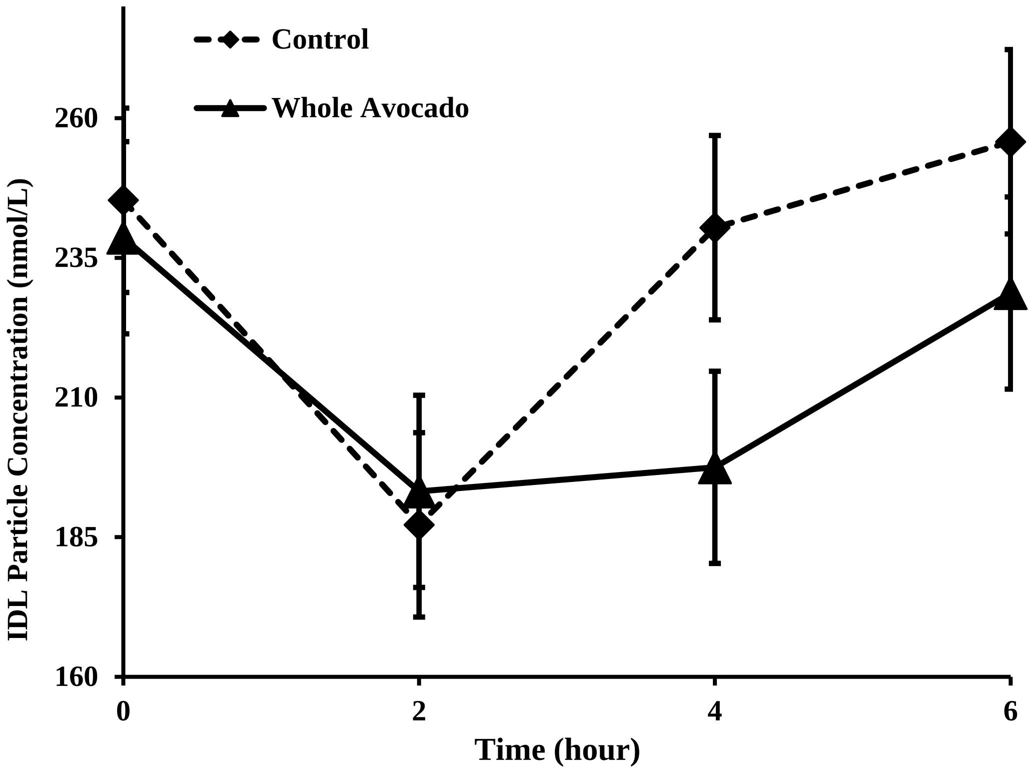

Figure S4. Plasma IDL particle concentrations over 6 h after consuming test breakfast meals (n=31). Data were analyzed by PROC MIXED using SAS 9.4. Main effects of meal *p* =0.004, time *p*=0.024, and meal by time p=0.013.

Data are means ± SE, n=31.

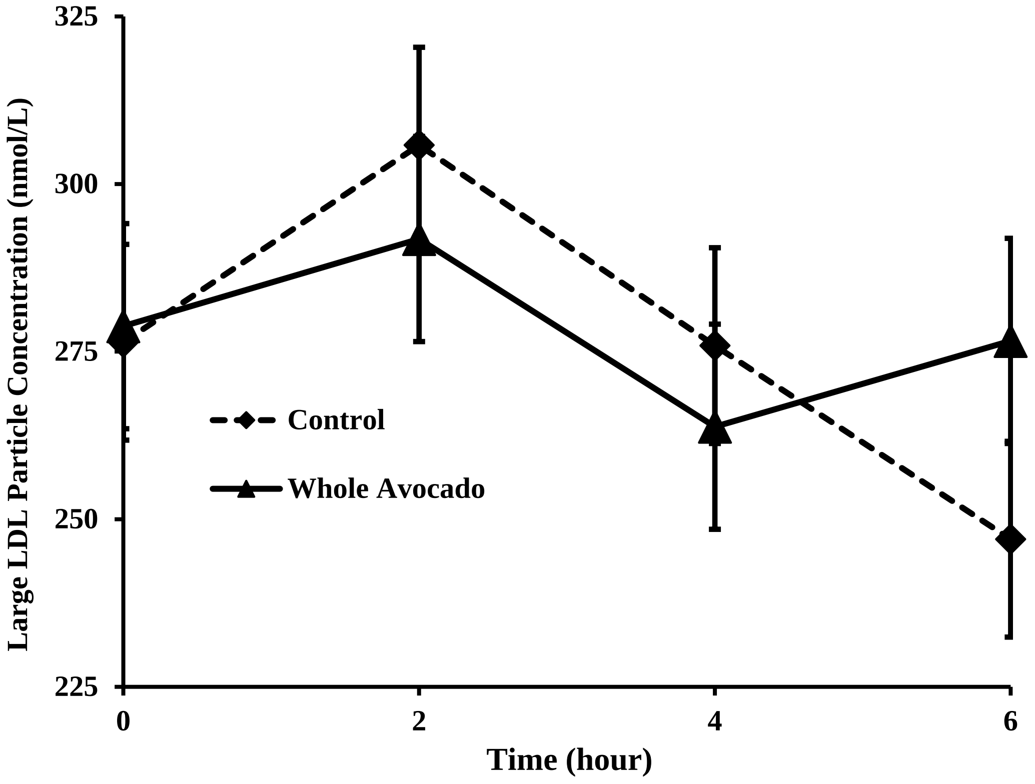

Figure S5. Plasma large LDL particle concentrations over 6 h after consuming test breakfast meals (n=31). Data were analyzed by PROC MIXED using SAS 9.4. Main effects of meal *p* =0.87, time *p*=0.032, and meal by time p=0.32.

Data are means ± SE, n=31.

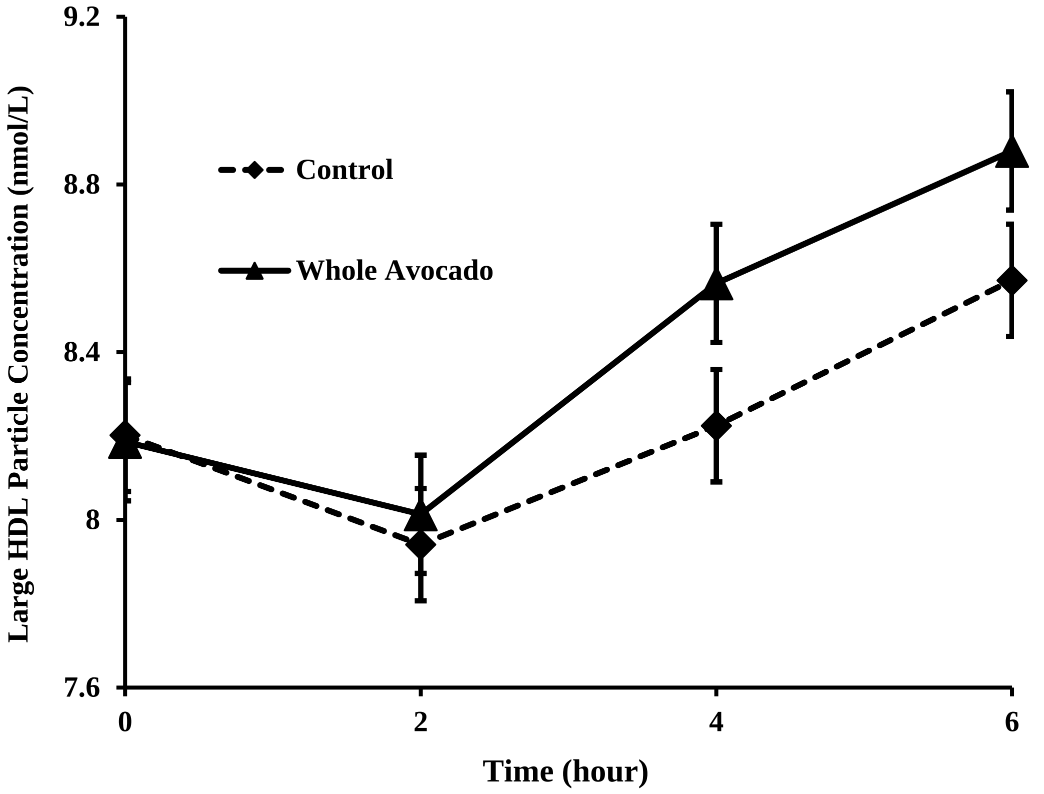

Figure S6. Plasma large HDL particle concentrations over 6 h after consuming test breakfast meals (n=31). Data were analyzed by PROC MIXED using SAS 9.4. Main effects of meal *p* =0.06, time *p*<0.0001, and meal by time p=0.43.

Data are means ± SE, n=31.

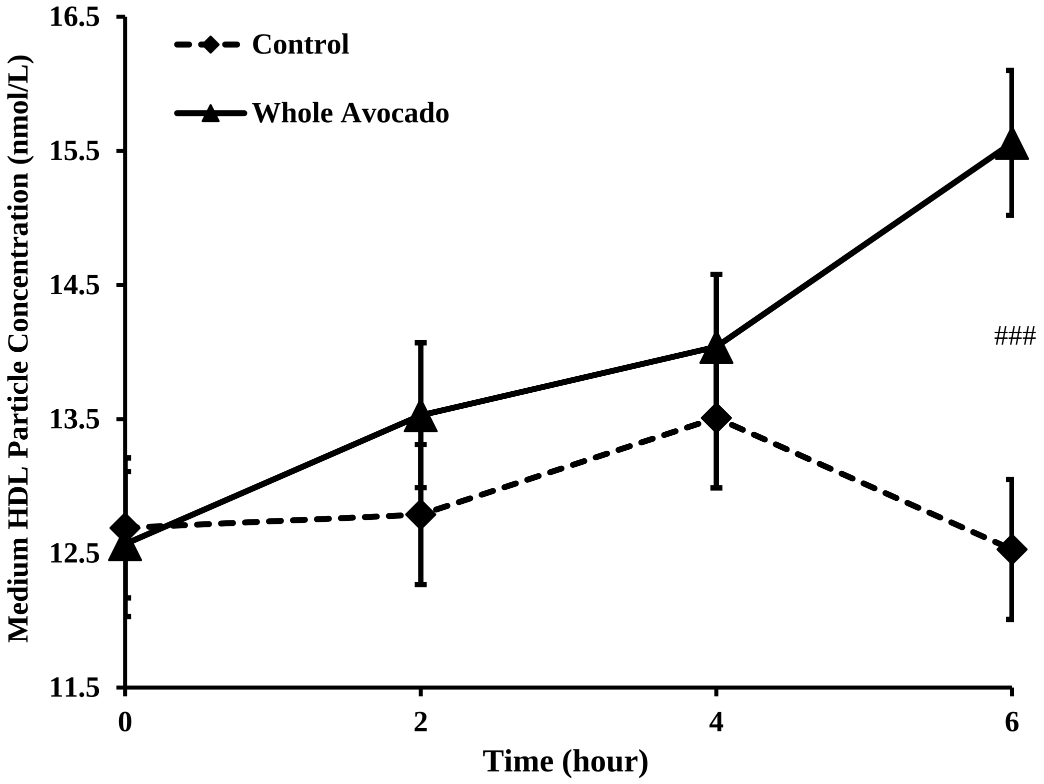

Figure S7. Plasma medium HDL particle concentrations over 6 h after consuming test breakfast meals (n=31). Data were analyzed by PROC MIXED using SAS 9.4. Main effects of meal p =0.004, time p=0.024, and meal by time p=0.013.

Data are means ± SE, n=31. ### p<0.0001 for the difference compared to the Control meal.

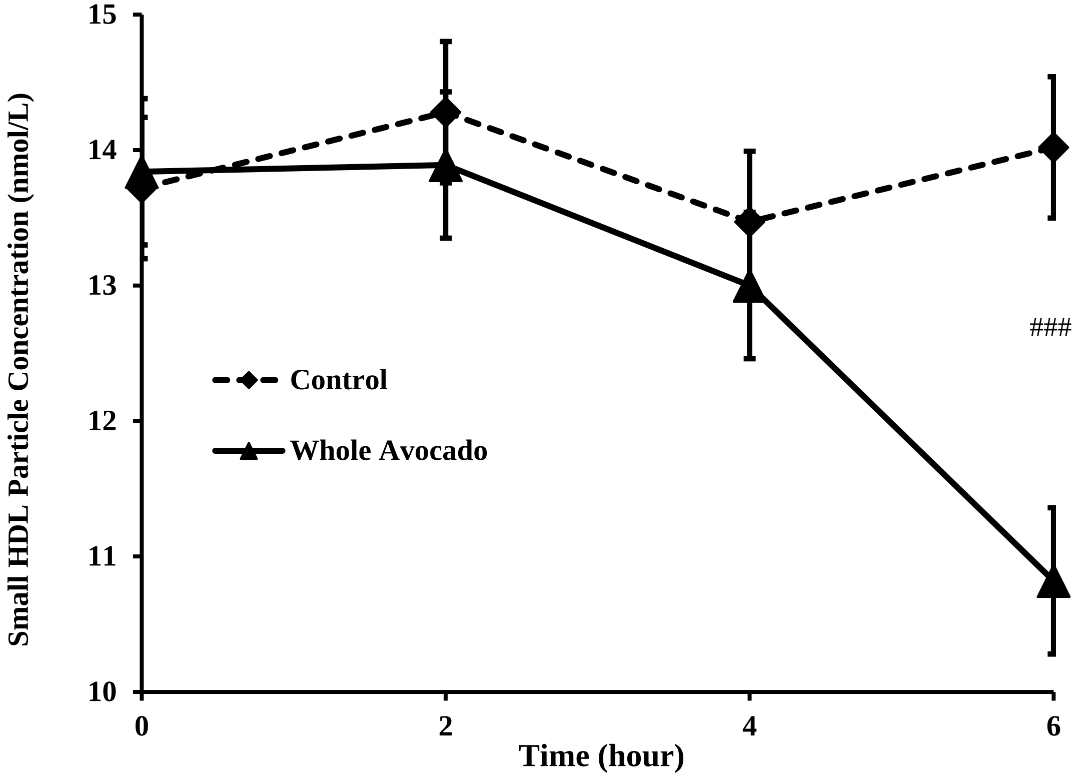

Figure S8. Plasma small HDL particle concentrations over 6 h after consuming test breakfast meals (n=31). Data were analyzed by PROC MIXED using SAS 9.4. Main effects of meal *p* =0.009, time *p*=0.011, and meal by time p=0.009.

Data are means ± SE, n=31. ### p<0.0001 for the difference compared to the Control meal.
